# Soldering Characteristics and Mechanical Properties of Sn-1.0Ag-0.5Cu Solder with Minor Aluminum Addition

**DOI:** 10.3390/ma9070522

**Published:** 2016-06-28

**Authors:** Yee Mei Leong, A.S.M.A. Haseeb

**Affiliations:** Centre for Advanced Materials, Department of Mechanical Engineering, Faculty of Engineering, University of Malaya, Kuala Lumpur 50603, Malaysia; leongyeemei@siswa.um.edu.my

**Keywords:** intermetallic, mechanical properties, nanoindentation, interfacial reaction, microstructure, differential scanning calorimetry, scanning electron microscopy, minor Al addition

## Abstract

Driven by the trends towards miniaturization in lead free electronic products, researchers are putting immense efforts to improve the properties and reliabilities of Sn based solders. Recently, much interest has been shown on low silver (Ag) content solder SAC105 (Sn-1.0Ag-0.5Cu) because of economic reasons and improvement of impact resistance as compared to SAC305 (Sn-3.0Ag-0.5Cu. The present work investigates the effect of minor aluminum (Al) addition (0.1–0.5 wt.%) to SAC105 on the interfacial structure between solder and copper substrate during reflow. The addition of minor Al promoted formation of small, equiaxed Cu-Al particle, which are identified as Cu_3_Al_2_. Cu_3_Al_2_ resided at the near surface/edges of the solder and exhibited higher hardness and modulus. Results show that the minor addition of Al does not alter the morphology of the interfacial intermetallic compounds, but they substantially suppress the growth of the interfacial Cu_6_Sn_5_ intermetallic compound (IMC) after reflow. During isothermal aging, minor alloying Al has reduced the thickness of interfacial Cu_6_Sn_5_ IMC but has no significant effect on the thickness of Cu_3_Sn. It is suggested that of atoms of Al exert their influence by hindering the flow of reacting species at the interface.

## 1. Introduction

Restriction on the use of lead based solder in the electronic industry has led to extensive developments in lead free solders. Driven by the necessity to improve the reliability of lead free electronic products and by the trend towards miniaturization, researchers are putting intense efforts into improving the properties of Sn based solders. Sn-Ag-Cu (SAC solder) alloys have been a favored replacement for Sn-Pb solders. However, the currently used ternary eutectic (T_m_ = 217 °C) or near eutectic SAC solders have some drawbacks. These SAC solders were reported to have relatively high amount of undercooling (10–30 °C), as β-Sn requires large undercooling to nucleate and solidify. This large undercooling promotes the formation of large, plate-like Ag_3_Sn structures that have been reported to cause joint embrittlement and reliability problem [1]. Research has been done on solders with lower silver content of, e.g., Sn-1.0Ag-0.5Cu (SAC105), in an attempt to inhibit the formation of Ag_3_Sn and also to reduce the solder cost, as the price of Ag has increased dramatically in recent years [2,3,4,5]. Although it has been reported that SAC105 performs better in drop tests, it has a higher liquidus temperature, which requires a higher reflow profile than the eutectic Sn-3.8Ag-0.7Cu (SAC387) alloy [5]. It also performs poorly in thermal cycling tests [6]. These drawbacks have prompted researchers to modify the solder content by adding small amounts of other alloying elements to further improve its reliability and lower its melting temperature.

Fourth minor alloying elements (0.05–0.1 wt.%) that have been added into SAC solder include Ni, Co, Fe, Mn, Zn, Ti, Ce, In and Al [7,8,9,10]. The effects of the fourth alloying element on the microstructure and mechanical properties have been investigated. Improvements in mechanical properties such as shear strength [10], tensile strength [7], impact resistance [5], and creep resistance [7] have been reported. It has been observed that the addition of fourth alloying elements imparts their influence on the mechanical properties by modifying the microstructure of the solder. It was observed that the fourth alloying element modify the microstructure, by: (i) refining the microstructure of the solder matrix; (ii) suppressing brittle intermetallic compound (IMC) formation; and (iii) changing the morphology of the IMC in solder matrix/interface [11].

The addition of aluminum has attracted much interest in recent years. Research on Al addition as nanoparticles or minor alloying element has been reported [12,13,14,15,16,17,18]. Amagai [12] observed that the addition of 0.05% Al nanoparticles did not have any significant effect on the interfacial IMC. As for the addition of aluminum as minor alloying element, a few works on the effect of Al on the mechanical properties, microstructure and solder/Cu substrate interfacial reaction have been reported [2,9,11,14,15,16,17,18]. Previous studies have shown that minor addition of Al gives promising results in suppressing undercooling of β-Sn and reduced formation of brittle phase Ag_3_Sn in SAC solder matrix [14]. It was found that Al addition (0.05 wt.%) results in excellent shear strength retention after thermal aging at 150 °C for up to 1000 h. Faizul et al. have investigated the effect of Al (0.1–2 wt.%) on the tensile strength of SAC105 [12,19]. They found that with 0.1 wt.% Al addition, Al reduced the yield strength and ultimate tensile strength (UTS) and promoted ductile fracture. When Al addition was more than 0.1 wt.%, the yield strength and ultimate tensile strength of the solder increased as a function of Al content and brittle fracture modes were seen.

A couple of studies dealt with the effects of Al on the bulk microstructure and interfacial IMC between solder and copper substrate [15,16,18]. With the addition of Al into Sn-Ag (SA) solder, Al_2_Cu IMC and ambiguous Al-Cu IMC were found in the bulk microstructure of SA solder on copper substrate after reflow [16,18]. On the other hand, only ambiguous Al-Cu IMC (Anderson et al. reported it as δ-Cu_33_Al_17_) was found in the bulk microstructure of SAC [14,19]. Though the reported effect of Al on the bulk microstructure seen by other researchers seems consistent, there are contradictory reports on the effects of Al addition on the interfacial solder/Cu reaction. With addition of 0.5 wt.% of Al into SA solder, Xia et al. observed a spalled layer of Al_2_Cu compound layer near the interface and the suppression of the growth of the Cu_6_Sn_5_ layer. When Al was added up to 1 wt.%, a layer of Al_2_Cu compound was formed at the solder/Cu interface, which completely replaced Cu_6_Sn_5_ layer [15]. On the other hand, Kotadia et al. reported that spherical Al-Cu IMC spalled away from the solder matrix when Al was depleted in the solder and there was no suppression of the growth of interfacial Cu_6_Sn_5_ layer when 0.5 to 2.0 wt.% of Al was added in to SA solder [18]. Li et al. have reported that with the addition of 1 wt.% Al into SAC387 solder, layered δ-Al_2_Cu_3_ formed at the interface after 10 min reflow and the growth of the Cu_6_Sn_5_ layer was reduced [16]. Dhaffer et al. have investigated the effect of 0.1.wt.% Al addition on the interfacial reaction by dipping method, they reported that 0.1% Al addition have suppressed the growth of Cu_6_Sn_5_ layer [20]. Until now, no detailed study has been done for the effect of minor Al addition (<1 wt.%) on interfacial reaction of SAC/Cu.

Nanoindentation as a mechanical testing method has attracted a great deal of attraction in many fields of research as it has the ability to measure the properties of sample in extremely small scales such as thin films and coating in nanometer range [21]. This technique is well suited to investigate the mechanical properties of IMCs, which have thicknesses of only several nanometers/micrometers. Cu-Al IMC was reported to form in SAC even with addition of as low as 0.05 wt.% [22]. Besides, it has been reported that Al is likely to substitute into Cu_6_Sn_5_ as well [14] Previous studies with addition such as Ni, and Mn have shown that solubility of minor alloying element in Cu_6_Sn_5_ could alter the nanomechanical properties of Cu_6_Sn_5_ [14]. Thus, understanding the mechanical properties of Cu-Al IMC and Cu_6_Sn_5_ is essential in the understanding of deformation behavior and failure mechanisms in lead-free solder joints.

The present work investigates the effect of Al on the interfacial IMC between SAC105 solder alloy and copper substrate. This work concentrates on the effect of the lower percentage of Al (0.1–0.5 wt.%) on the Cu–Sn reaction during reflow and isothermal aging. As has been noted earlier [15,16], absence of Cu in the solder led to the formation of Al_2_Cu and Al-Cu IMC near/at the interface during reflow, and the IMCs tended to spall away from the solder matrix when Al was depleted in solder, thus complicating the situation at the interface. With the presence of Cu in SAC solder, it is expected that Al will react with Cu in the bulk and thus provide a more simplified scenario at the interface, which may lead to a better understanding of the effects of Al. This study concentrates on the lower percentage of Al (0.1%–0.5%), as higher percentages may lead to formation of Al_2_Cu and other IMCs at the interface. Furthermore, this study investigates the nanomechanical properties of Cu-Al IMC particles and also examines whether addition of Al would affect the nanomechanical properties of Cu_6_Sn_5_.

## 2. Results

### 2.1. Differential Scanning Calorimetry

**Figure 1:**
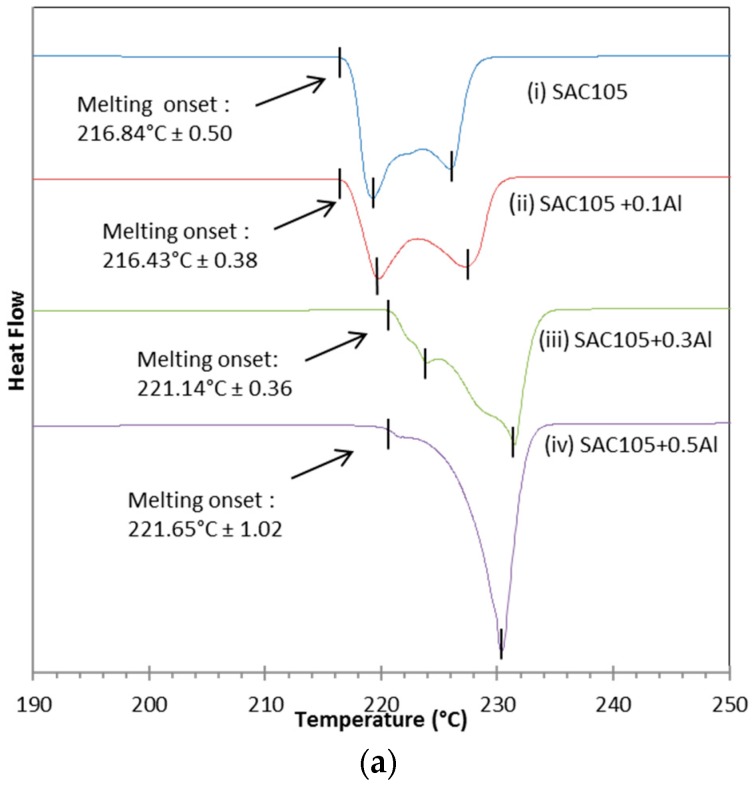

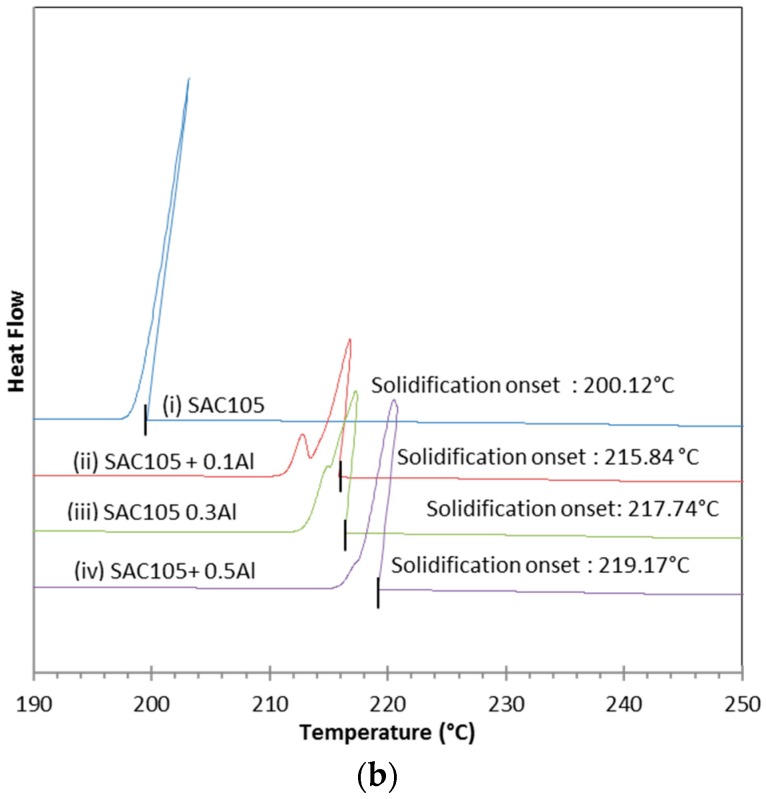
DSC curves for as cast SAC105, SAC105 + 0.1Al, SAC105 + 0.3Al and SAC105 + 0.5Al alloys: (**a**) heating; and (**b**) cooling.

Figure 1 shows the Differential Scanning Calorimetry (DSC) curves for SAC105, SAC105 + 0.1Al, SAC105 + 0.3Al and SAC105 + 0.5Al. During heating, SAC105 and SAC105 + 0.1Al solder show an onset melting temperature (T_m_) of 216.84 °C ± 0.50 and 216.43 °C ± 0.38, respectively. This onset temperature corresponds to the ternary eutectic reaction (T_m_ = 217 °C) of Sn-Ag-Cu alloy, L → Ag_3_Sn + Cu_6_Sn_5_ + Sn [23]. Two prominent peaks were seen at ~221 °C and ~228 °C in SAC105 and SAC105 + 0.1Al (Figure 1a,b). These two peaks were associated with the eutectic temperature for Sn-Ag (L → Ag_3_Sn + Sn) and Sn-Cu (L → Cu_6_Sn_5_ + Sn), respectively. As the amount of Al was added up to 0.3 wt.%, there is a change in the DSC curve (Figure 1c). The onset temperature was shifted to 221.14 °C ± 0.36 and 221.65 °C ± 1.02, respectively for SAC105 + 0.3Al and SAC105 + 0.5Al which corresponded to Sn-Ag eutectic temperature (T_m_ = 221 °C). For SAC105 + 0.3Al, a first small peak of heat absorption appears at 224 °C, followed by a second larger peak of heat absorption at ~231 °C (Figure 1c). For SAC105 + 0.5Al, only a large peak of heat absorption appears at ~231 °C (Figure 1d). The peak at ~231 °C corresponds to the melting temperature of Sn (T_m_ = 231 °C).

During cooling, the nucleation temperature was determined by the onset solidification of the exothermic peak (Figure 1b). SAC105 has an onset solidification at 200.12 ± 0.64 °C. Addition of aluminum to SAC105 shifted the exothermic peak to the right, where the onset solidification temperature is 215.84 °C ± 1.39, 217.74 °C ± 0.83 and 219.17 °C ± 0.21 for SAC105 + 0.1Al, SAC105 + 0.3Al and SAC105 + 0.5Al, respectively.

The degree of undercooling, ∆T, was defined by the difference of two onset temperatures in cooling and heating curve. In Table 1, it can be seen that SAC105 has the largest undercooling of ~17 °C. The addition of Al into SAC105 solder reduced the degree of undercooling significantly in all cases where Al was added.

### 2.2. Microstructure

**Figure 2:**
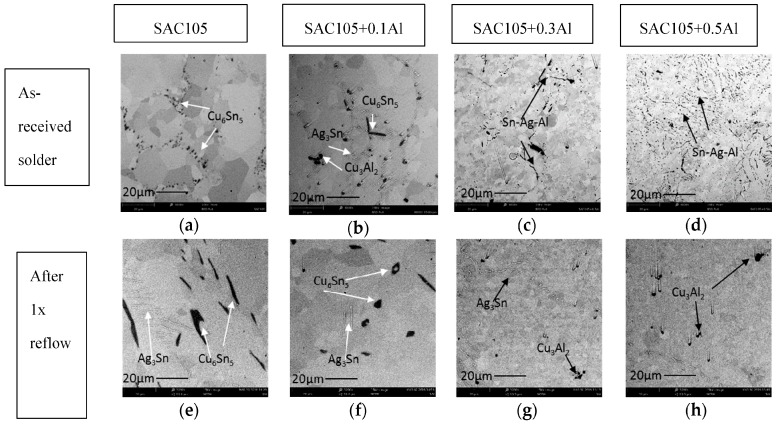
SEM images of cross sectional samples of bulk microstructure of as cast (**a**–**d**) and after 1 × reflow (**e**–**h**) of SAC105, SAC105 + 0.1Al, SAC105 + 0.3Al and SAC105 + 0.5Al.

Figure 2a–h shows the cross sectional Scanning Electron Microscopy (SEM) images of as-received solder alloys and reflowed solder joints prepared on copper substrates. Cross sectional images of as-received SAC105 samples show primary Sn phase having a lighter contrast while the darker contrast phase represents Cu_6_Sn_5_. The Cu_6_Sn_5_ formed a continuous network in the as-cast SAC105 solder and fine Ag_3_Sn particles are also seen in the as-cast solder. With the addition of 0.1 wt.% Al, Cu_6_Sn_5_ phase and particles of another new darker phase are seen distributed in the lighter contrast Sn phase. The darker particles are seen to agglomerate in the Sn phase (Figure 2b). With the addition of 0.3 wt.% and 0.5 wt.% Al, elongated shapes of new darker IMC phase are seen distributed in the lighter contrast Sn phase. It can be seen that the Sn grain size decreases with Al addition in the as-received alloys. After reflow, the bulk microstructure of the reflowed solder was different from that of the as-received solder. From Figure 2e,f it can be seen that Cu_6_Sn_5_ and Ag_3_Sn particles are larger after 1 × reflow as compared to Figure 2a,b. After 1 × reflow, with the addition of 0.3 wt.% and 0.5 wt.% Al, equiaxed darker IMC was seen distributed in Sn phase instead of elongated shape of new darker IMC in the as-received sample. Sn grain size reduction as a function of Al content still persists in reflowed sample.

**Figure 3:**
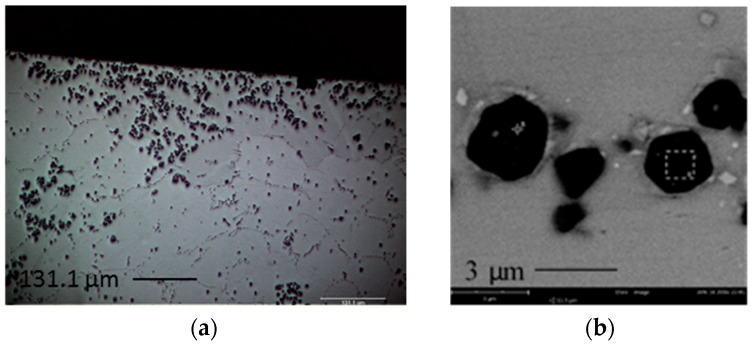
(**a**) Optical microscope cross sectional images SAC105 + 0.5Al near top surface of the solder after 1 × reflow and (**b**) FESEM images of equiaxed but faceted IMCs found at cross-sectioned of SAC105 + 0.5Al.

Figure 3a shows the optical microscope cross-sectional images of SAC105 + 0.5Al near top surface of solder after 1 × reflow. It can be seen that the new darker phase equiaxed IMC agglomerated and was mostly found near the top surface of the solder joint. Agglomeration of the new darker phase near the top surface of solder is also found SAC105 + 0.3Al. Figure 3b shows the new darker phase equiaxed IMC under high magnification. It can be seen that the new faceted IMC particles have varied sizes, ranging from 1 to 5 μm. 

**Figure 4:**
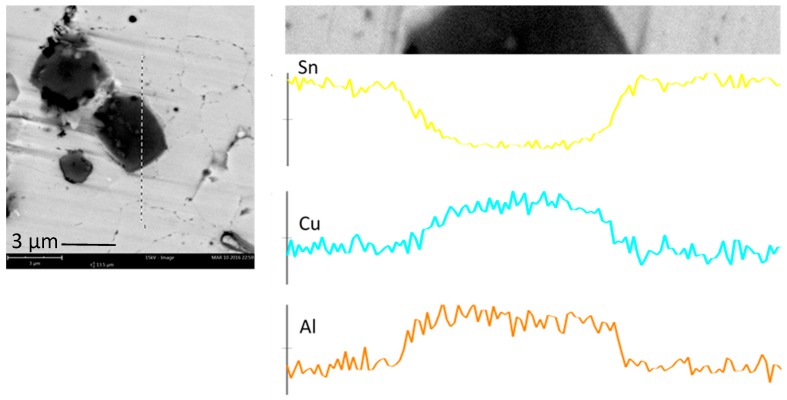
EDS line scan across equiaxed IMCs in SAC105 + 0.5Al.

**Figure 5:**
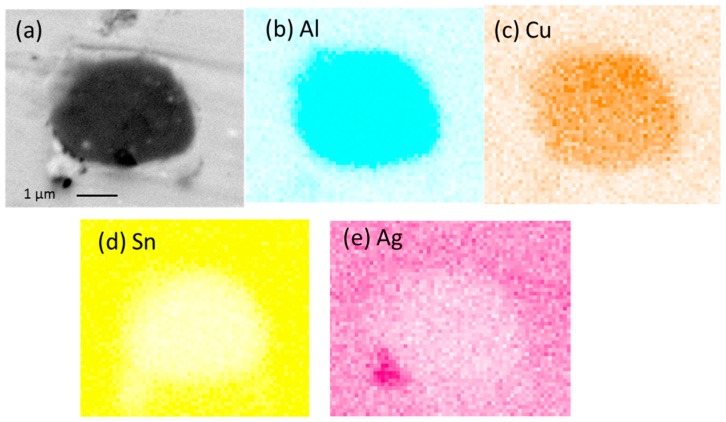
(**a**) Cross-sectioned image of SAC105 + 0.5Al after 1 × reflow. Elemental maps for the constituent elements: (**b**) Al; (**c**) Cu; (**d**) Sn; and (**e**) Ag.

Energy-dispersive X-ray Spectroscopic (EDS) analysis was conducted on the new IMC phase found in SAC105 + 0.1Al, SAC105 + 0.3Al and SAC105 + 0.5Al. Analysis results indicated that the composition of the new phase was 60–65 at.% Cu, 35–40 at.% Al. EDS Line scan and elemental mapping analysis were also conducted on the new IMC phase (Figure 4 and Figure 5). Both analysis confirmed that this darker IMC phase consists of only Al and Cu. Based on the ratio of Al and Cu content, possible identification of this darker IMC phase is Cu_3_Al_2_ (δ) or Cu_9_Al_4_ (γ_1_), both of which could exist in the temperature range below 300 °C [24].

In the previous studies on minor addition of Al, many researchers attempted to identify Cu-Al IMC by EDS analysis. There are a few Cu-Al binary phases that have compositions close to each other, such as γ_1_-Cu_9_Al_4_, ζ_2_-Cu_4_Al_3_, and δ-Cu_3_Al_2_. This makes it difficult for researchers to accurately identify the exact Cu-Al phase form [2,17,20]. Similarly, in this study, EDX analysis could not provide clear identification for the Cu-Al compound. Anderson et al. who studied the effect of Al addition on the microstructure of solder have identified the Cu-Al IMC as δ-Cu_33_Al_17_. They did so by casting out a Cu-Al block which has the similar composition with the Cu-Al IMC they obtained in the bulk solder, and analyzed it using X-ray diffraction (XRD) [14]. Though Anderson et al. have chosen to refer to it as δ-Cu_33_Al_17_, the general nomenclature for these IMC is also known as δ-Cu_3_Al_2_ [24]. By comparing the morphology of Cu-Al IMC (Figure 3a) in this study to δ-Cu_33_Al_17_ [14], it can be seen that they both exhibit a similar morphology (equiaxed and faceted dark color appearance under Field-emission scanning electron microscopy (FESEM) and size (size varied from 1 to 5 μm)). Thus, it would be logical to assume both IMC are of the same kind, and it will be referred to as Cu_3_Al_2_ in the following discussion.

### 2.3. Interfacial Reaction after Reflow

**Figure 6:**
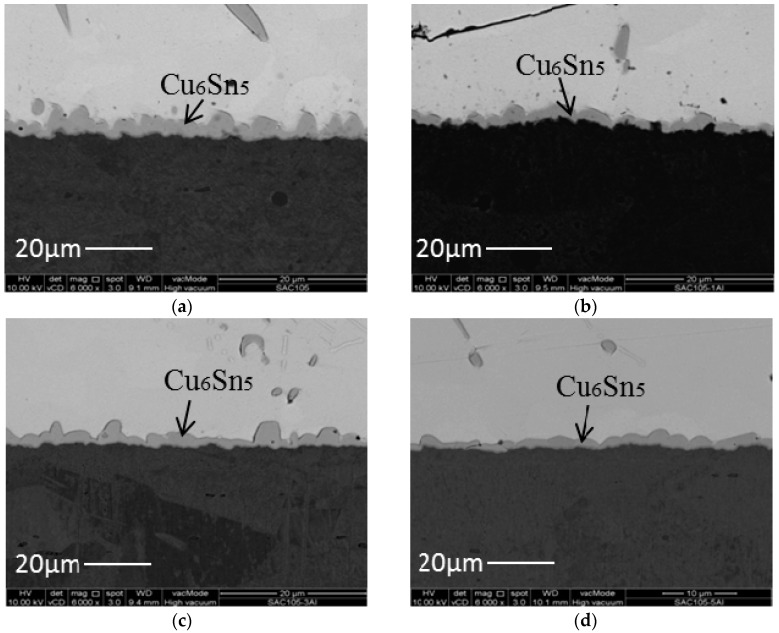
Cross sectional FESEM micrographs of: (**a**) SAC105; (**b**) SAC105 + 0.1Al; (**c**) SAC105 + 0.3Al; and (**d**) SAC105 + 0.5Al after 1 × reflow.

**Figure 7:**
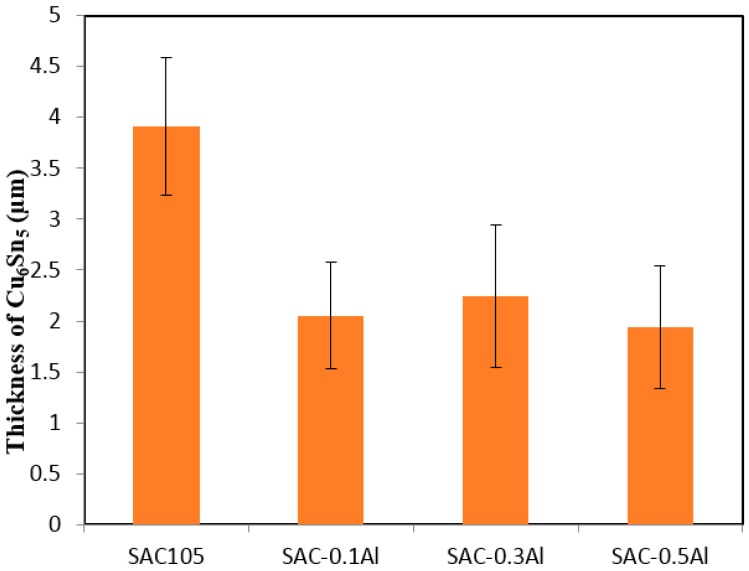
Variation of Cu_6_Sn_5_ thickness with Al content during 1 × reflow.

Figure 6 shows cross-sectional FESEM micrographs at the solder/Cu interface after 1 × reflow. A typical scallop type Cu_6_Sn_5_ layer forms at the SAC105/Cu interface after reflow (Figure 6a). Upon the addition of aluminum, the scallop morphology is still seen, but the IMC becomes more flat as Al is added up to 0.5 wt.% (Figure 6c). The IMC height is reduced with the addition of Al. The average thickness of the total IMC layer is plotted as a function of Al content in Figure 7. The influence of Al addition on the interfacial IMC thickness is obvious. The thickness of the interfacial IMC does not seem to vary with the Al content of the solder in the range 0.1–0.5 wt.%. This may indicate that the addition of Al beyond a certain percentage does not bring additional benefit in terms of suppression of IMC growth. Cu_6_Sn_5_ was the only IMC found at the interface, and no trace of Al in Cu_6_Sn_5_ and Al-Cu compound could be detected at the interface of all aluminum-added solder.

### 2.4. Top View of Cu_6_Sn_5_ Solder after 1 × Reflow

**Figure 8:**
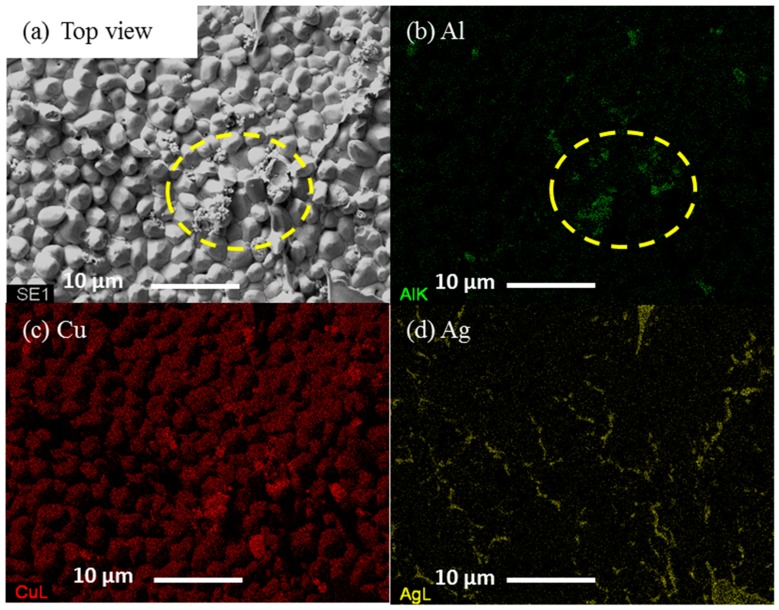
(**a**) Top view image of deeply etched SAC105 + 0.5Al after 1 × reflow. Elemental maps for the constituent elements: (**b**) Al; (**c**) Cu; and (**d**) Ag.

Figure 8a shows top view image of deeply etched SAC105 + 0.5Al after 1 × reflow. Prior to FESEM imaging, both samples were deeply etched in a mixture of 93% CH_3_OH, 5% HNO_3_ and 2% HCl to remove the solder matrix and thereby expose the interfacial IMC. The IMC grains found on the interface of SAC105 and SAC105 + 0.5Al were identified as Cu_6_Sn_5_. It can be seen from the images that SAC105 + 0.5Al shows somewhat faceted Cu_6_Sn_5_ grains. Under high magnification in the high Al concentration region, small and agglomerated particles are seen (encircled in yellow dotted line). EDS analysis was done on the high Al concentration particle. Figure 8b–d shows the EDS elemental maps. Under high resolution imaging and elemental mapping, Al, Ag and Cu were detected on the surface of the exposed IMC. In Figure 8b, the presence of Al is indicated in green. The region of high concentration of Al is encircled in yellow. It can be seen that the region with high Al concentration corresponds to that of Cu (compare Figure 8b,c). The agglomerated particles are identical to the Cu-Al IMC that was found in the bulk microstructure. The elongated and plate-like particles (right corner of Figure 8d) on the Cu_6_Sn_5_ IMC grains are identified as Ag_3_Sn.

### 2.5. Interfacial Reaction After Isothermal Aging

**Figure 9:**
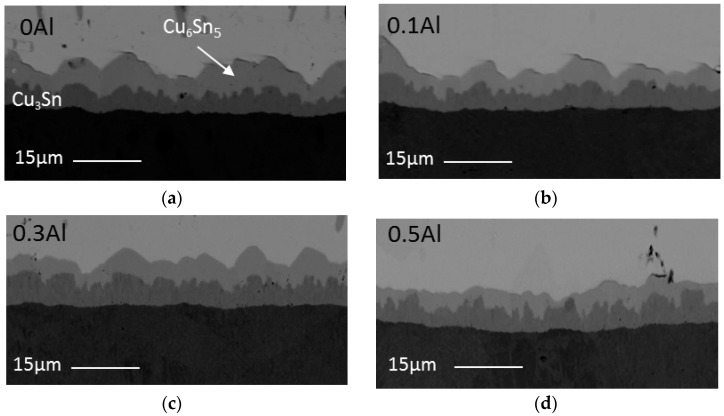
Cross sectional images of: (**a**) SAC105; (**b**) SAC105 + 0.1Al; (**c**) SAC105 + 0.3Al; and (**d**) SAC105 + 0.5Al after aging at 150 °C for 720 h.

After 1 × reflow, some of the solder samples were also subjected to isothermal aging at 150 °C for up to 720 h in order to study the effects of Al addition on the solid state reaction between solder and copper substrate. Figure 9 shows the cross sectional images of isothermally aged SAC105, SAC105 + 0.1Al, SAC105 + 0.3Al and SAC105 + 0.5Al. After thermal aging for 720 h, another intermetallic layer (darker layer) formed in between the first intermetallic layer and Cu substrate in both SAC and SAC + Al solders. EDS was used to determine the composition of each layer. It is confirmed by elemental ratio by EDS analysis that the outer layer is Cu_6_Sn_5_ and the inner layer is Cu_3_Sn. Within the resolution of EDS, there was no aluminum detected in both Cu_6_Sn_5_ and Cu_3_Sn after thermal aging. The morphology of Cu_6_Sn_5_ and Cu_3_Sn are seen to be similar in SAC105 and SAC105 + Al solders.

**Figure 10:**
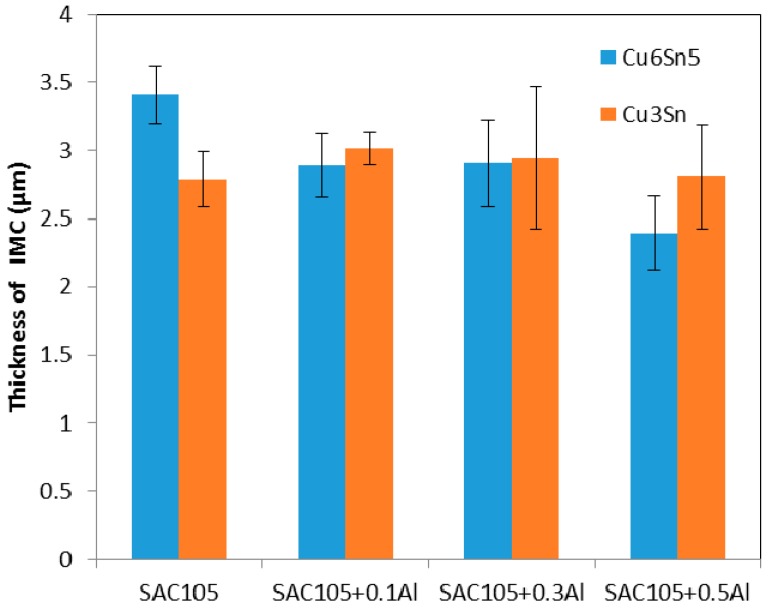
Variation of thickness of IMC with Al content after aging at 150 °C for 720 h.

From Figure 9, it is seen that the addition of Al up to 0.5 wt.% has slowed down the growth of the total interfacial IMCs. Figure 10 shows the thickness of Cu_6_Sn_5_ and Cu_3_Sn plotted as a function of Al content. It can be seen that Cu_6_Sn_5_ is reduced as Al is added, up to 0.5 wt.%. However, the thickness of Cu_3_Sn is almost the same for all samples.

### 2.6. Mechanical Properties

**Figure 11:**
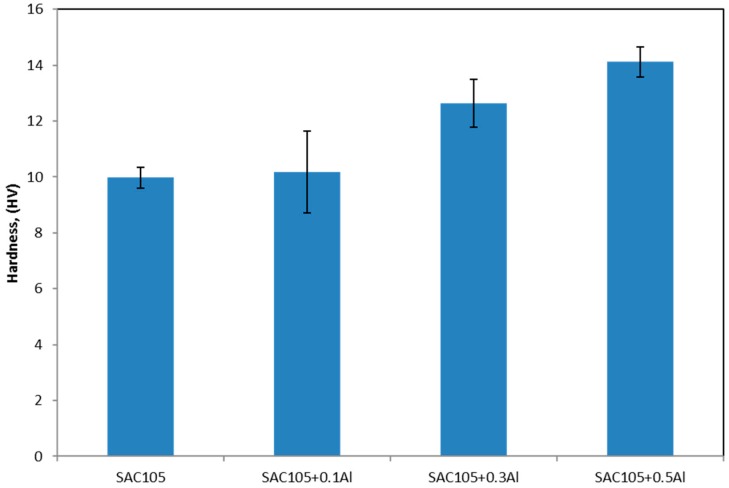
Variation of Vickers hardness with Al content for as received samples.

Indentation testing was carried out on bulk solder and the intermetallic phase in the solders, e.g., Cu_6_Sn_5_ and Cu_3_Al_2_. The hardness of the bulk solder was determined by Vickers hardness test while the hardness of individual intermetallic phase was determined by nanoindentation. Figure 11 shows the hardness of solder with varying Al content. It can be seen that the hardness of the solder increases as a function of Al content. SAC105 exhibited hardness of 9.78 HV while SAC105 + 0.5Al exhibited the highest hardness value of 14.12 HV. The result of SAC105 is in good agreement with other studies [25,26].

**Figure 12:**
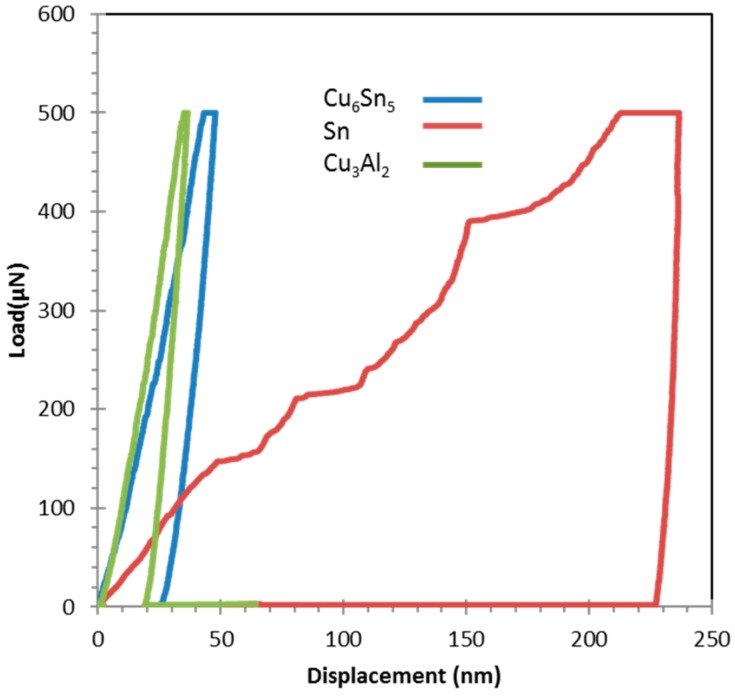
Load displacement data obtained for 500 µN maximum load indentations performed on Sn, Cu_6_Sn_5_ and Cu_3_Sn.

**Figure 13:**
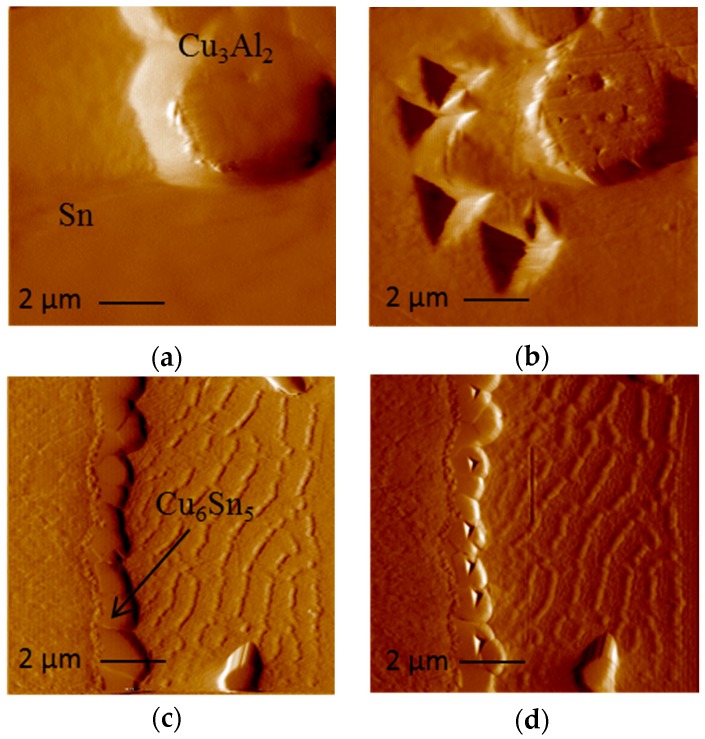
Scanning probe microscopy image of: (**a**) Sn and Cu_3_Al_2_ before indentation; (**b**) Sn and Cu_3_Al_2_ after indentation; (**c**) Cu_6_Sn_5_ before indentation; and (**d**) Cu_6_Sn_5_ after indentation of SAC105 + 0.1Al.

Figure 12 shows typical load displacement data obtained through indentations performed on Sn, Cu_6_Sn_5_ and Cu_3_Al_2_ of a maximum load of 500 µN. For a test of the same maximum load, the maximum penetration of the indenter for Sn-Ag-Cu solder is approximately 4.5 times of that measured for Cu_6_Sn_5_ and Al_3_Cu_2_. The solder matrix, as expected, is very soft, with a hardness of 0.23–0.3 GPa. The matrix exhibited significant plasticity. Upon unloading, the solder recovers only approximately 10 nm of the 230 nm that the indenter penetrated. In contrast to the solder, both intermetallics are significantly harder: Cu_6_Sn_5_ (~6.2 GPa) and Al_3_Cu_2_ (~10.50 GPa). The intermetallics typically recover around 40% of the maximum penetration of the indenter upon unloading. From this, the deformation of the intermetallic phases was found to be both elastic and plastic, while the deformation of the solder is found to be primarily plastic [26]. Scanning probe microscopy (SPM) was used to accurately perform indentation on specific IMC phases. It was also used to observe the residual indents, as shown in Figure 13. As expected from the nanoindentation data in Figure 12, the residual indents in the solder were much larger than in intermetallics (Figure 13). All of the residual indents observed for the intermetallics exhibited a smooth profile with no detected pile-up or sink-in of material, while softer materials like Sn exhibit a pile-up behavior.

**Figure 14:**
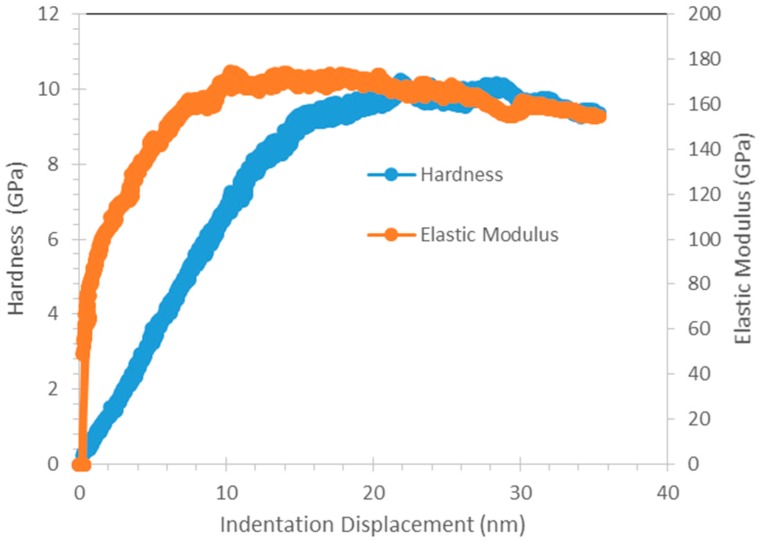
Hardness and Elastic Modulus of Cu_3_Al_2_ plotted against indent displacement.

Hysitron nanoDMA with CMX correction and maximum load of 1000 µN was used to perform continuous nanoscale dynamic mechanical measurement of Cu_3_Al_2_. Hardness and elastic modulus of Cu_3_Al_2_ could be obtained as a function of indentation depth. Figure 14 shows the hardness and elastic modulus of Cu_3_Al_2_ plotted against indent displacement. The hardness and elastic modulus of Cu_3_Al_2_ was lower near the surface but was fairly constant at depths greater than ~15 nm. The results are similar to that obtained from quasi-static measurement with maximum load of 500 µN.

As seen in Table 2, Sn has hardness and elastic modulus of 0.22–0.36 GPa and ~50 GPa, respectively, while Cu_6_Sn_5_ has hardness and elastic modulus of ~6.2 GPa and ~92–110 GPa, respectively. The results are in good agreement with other studies [27,28,29]. On the other hand, Cu_3_Al_2_ exhibits a much higher hardness and elastic modulus as compared to Sn and Cu_6_Sn_5_, with a hardness of ~10.50 GPa and an elastic modulus of ~170.08 GPa.

## 3. Discussion

From the DSC curves (Figure 1), it is found that when 0.1 wt.% of Al was added to the solder, the endothermic curve remains similar to that of SAC105. Both SAC105 and SAC105 + 0.1Al exhibit twin peaks endothermic curve. The onset melting temperature of SAC105 + 0.1Al is the same as that of SAC105, which is ~217 °C. This corresponds to Sn-Ag-Cu ternary eutectic temperature. When the percentage of Al is increased to 0.3 wt.% and 0.5 wt.%, the onset of melting temperature shifts to ~221 °C (Sn-Ag eutectic temperature) [30]. The DSC curves of SAC105 + 0.3Al and SAC + 0.5Al solders are similar to the DSC curve of non-eutectic Sn-Ag solder [31]. This shows that free Cu atoms are not available in SAC105 + 0.3Al and SAC + 0.5Al solders as Sn-Ag-Cu solder alloy always shows the onset melting at ~217 °C [32]. One possible explanation for this is the formation of Cu_3_Al_2_ compound, which has reduced the available Cu in the bulk solder to react with Sn and Ag. The Cu_3_Al_2_ compound already formed in the as-received samples, which were supplied in the as-cast condition. As seen in Figure 2b–d, Cu_3_Al_2_ is formed in the as-cast solder. Cu_3_Al_2_ has a higher melting temperature of 960 °C [33]. Thus, during the melting of solders for up to 300 °C, the stable Cu_3_Al_2_ IMC did not react and this lowers the activity of Cu in solder melts. The lack of free Cu atom in the solder is further indicated by the shift of a prominent peak in the DSC curves of SAC105 and SAC105 + 0.1Al. The ~228 °C (Sn-Cu eutectic temperature) peak in SAC105 and SAC105 + 0.5Al has shifted to ~231 °C (T_m_ of Sn = 232 °C) in SAC105 + 0.3Al and SAC105 + 0.5Al. This shift indicates that the deficiency of Cu atom in SAC105 + 0.3Al and SAC105 + 0.5Al has reduced the strong eutectic reaction of Sn-Cu, L → Cu_6_Sn_5_ + Sn. With Al addition, the peak indicating Sn melting is seen, as fractions of un-melted Sn remaining in the solder melted when temperature increased to 232 °C [30].

Table 1 shows that SAC105 has the largest undercooling of ~17 °C. This is well within range of undercooling values, 10–30 °C reported for Sn-Ag-Cu solder [1]. This is because β-Sn requires large undercooling to induce nucleation and solidification [1]. The presence of Al in SAC105 has reduced the undercooling significantly. It is seen that with addition of Al up to 0.5%, undercooling has reduced to 1–5 °C. Kotadia et al. and Anderson et al. have observed the effect of Al in reducing undercooling of Sn-3.5Ag and Sn-3.5Ag-0.95Cu solder to 7 °C and 4 °C respectively [14,34]. The addition of minor alloying element into solder has been one of the effective ways of reducing undercooling by promoting nucleation of β-Sn. Minor alloying atoms which have a much higher melting temperature can exist in molten Sn and provide heterogeneous site for β-Sn nucleation. In the case of Al as minor alloying, Cu_3_Al_2_ compound is formed even when the addition of Al is as low as 0.1%. During exothermic reaction in DSC, the existing Al-Cu intermetallic compounds act as a preferential site to promote heterogeneous nucleation of β-Sn, and thus lowering the undercooling of SAC105.

Segregated Cu_3_Al_2_ IMC was found near the top surface of the sample with addition of Al after 1 × reflow. One of the possible reasons could be that, during reflow, solders are melted on the Cu substrates at 250 °C, the Cu_3_Al_2_ particles are not wet by the molten solder, thus they are not drawn into the melt as the molten solder particles solidify. This causes rejection of the Cu_3_Al_2_, which remain near the surface/edges of the solder after reflow. Another possible reason for the segregation of Cu_3_Al_2_ particles at the edge/surface could be attributed to the buoyancy effects. The density of Cu_3_Al_2_ particles is 6.278 g/cm^3^, which is lower than the density of liquid Sn, 6.99 g/cm^3^ [35]. Thus, during reflow, the less dense Cu_3_Al_2_ particles migrate to the surface/edges. Kotadia et al. have reported the segregation of Al_2_Cu in SA solder. They suggested that the segregation of Al rich phase and Al-Cu compound is caused by Stokes and Marangoi motion, which is due to large stable liquid miscibility gap in binary Sn-Al and ternary Sn-Ag-Al [18].

Minor alloying elements which could affect the growth of Cu-Sn compound are divided into two categories: (i) elements that show marked solubility in either one or both of the Cu-Sn IMCs; and (ii) elements that do not significantly dissolve in either of the Cu–Sn IMCs [11]. The effect of elements in category 1 on IMC growth could be explained by using thermodynamic argument. These elements stabilize Cu_6_Sn_5_ and decrease the growth of Cu_3_Sn. The elements in category 2 do not have a prominent effect on IMC as they only affect the growth of IMC layers indirectly. It can be seen from Figure 4 and Figure 5 that the addition of Al has reduced the growth of Cu_6_Sn_5_. Though Anderson et al. have suggested Al has some solubility in Cu_6_Sn_5_, no trace of Al could be found in Cu_6_Sn_5_ in this study. In this study, the addition of up to 0.5 wt.% Al did not alter the scallop morphology of Cu_6_Sn_5_ [14]. Scallop type IMC formation is promoted by higher value of the IMC/liquid solder interfacial energy.

There is very limited amount of information available on the influence of Al on the Cu–Sn reaction. Li et al. have reported the suppression of Cu_6_Sn_5_ IMC growth with addition of 1% Al. They suggested that the suppression is due to the formation of an Al-Cu IMC layer at the interface, which acts as a barrier for Cu and Sn diffusion [16]. However, Al-Cu IMC layer is not found at the interface in this study as the amount of Al added is less (≤5%). Dhaffer et al. have also reported suppression of Cu_6_Sn_5_ IMC growth with addition of 0.1% Al during dip soldering and solid state reaction [20]. The Cu_3_Al_2_ IMC found at the Cu_6_Sn_5_/Sn interface (Figure 8) could account for the suppression of Cu_6_Sn_5_. This is seen in previous work where Zn found at the Cu_6_Sn_5_/Sn interface, hindered the flow of copper atoms to the solder thereby slowing down the IMC growth [36]. Thus, the segregation of Al atoms at the IMC/Sn interface may have similar effect on the growth of IMC, by hindering the flow of Cu or Sn atom. With minor Al addition, most of the Al reacts with Cu in the bulk solder to form Cu_3_Al_2_. Hence, Al does not form a layer of compound at the interface. Reduction of free Cu atom in the bulk solder could also be attributed to the retardation of Cu_6_Sn_5_ growth. When the amount of Al in the solder increased (≥0.5 wt.% Al in SA, ≥1 wt.% Al in SAC), it formed a layer of Cu-Al compound at the solder/Cu interface [15,16]. By their presence at the interface, Cu_3_Al_2_ IMC hinders the flow of Cu or Sn atom to the solder thereby retarding IMC growth during reflow.

During isothermal aging, the Cu_6_Sn_5_ IMC layer grows by interdiffusion of Cu and Sn and reaction with each other, while the Cu_3_Sn IMC forms and grows by reactions between the Cu substrate and Cu_6_Sn_5_ IMC layer, as given in the equation below [11]:

Cu_6_Sn_5_ + 9Cu → 5Cu_3_Sn (1)

The presence of Cu_3_Al_2_ IMC at the Cu_6_Sn_5_/Sn interface hinders the flow of Cu or Sn atom to the solder, however it does not affect the reaction in Equation (1). Cu_3_Sn grows by consuming Cu_6_Sn_5_ that is formed during reflow. Thus, the thickness of Cu_3_Sn was not significantly affected by the addition of Al in solder. On the other hand, with slower interdiffusion of Cu and Sn at the interface (due to presence of Cu_3_Al_2_) and Cu_3_Sn formation by consumption of Cu_6_Sn_5_, the thickness of Cu_6_Sn_5_ was reduced in SAC105 + Al solder.

The addition of Al has increased the hardness of bulk solder. This could be due to the grain refinement of Sn, which can be seen in Figure 3. Kim et al. has also reported that addition of Al as low as 0.01 wt.% could refine the Sn grain of Sn-Cu solder [8]. From nanoindentation, Cu_3_Al_2_ that are found in all SAC + Al solder exhibits higher hardness than other elements in SAC105 + Al. This seems promising in strengthening the solder joints. However, its high elastic modulus should be considered as well, as high elastic modulus could be detrimental for impact testing. Thus, further testing need to be done to further verify the effects of strengthening effects of Cu_3_Al_2_.

In spite of the incorporation by only a small fraction, Al is clearly seen to have influenced decisively on the hardness, undercooling of solder and the interfacial characteristics during reflow. However, the amount of Al must be kept below 0.3 wt.% in order to avoid large Cu_3_Al_2_ agglomeration, which could affect the performance of solder.

## 4. Materials and Methods

SAC105-xAl (where x = 0, 0.1, 0.3, 0.5 wt.%) were fabricated by Beijing Compo Advanced Technology Co. Ltd. (Beijing, China). All the solder alloys were prepared in rod shape (0.7 cm diameter, 15 cm length). The solder alloy was then cut into thin disks with 1 mm thickness by using electric discharge machining (EDM, A500W, Sodick, Schaumburg, IL, USA).

As-received solder was prepared for micro-examination by standard metallographic technique which included, cutting, mounting, grinding (up to 3000 grit paper) and polishing (diamond paste with size 9 µm, 6 µm, 3 µm, 1 µm and colloidal silica with size 0.2 μm). The microstructure of the as-received SAC105-Al solders was characterized by Field emission scanning electron microscope (FESEM, FEI-FEG450, FEI, Houston, TX, USA) and intermetallic compound (IMC) composition was investigated by energy dispersive X-ray spectroscope (EDS, EDAX-Genesis Utilities, EDAX, Mahwah, NJ, USA). Differential scanning calorimeter, DSC (DSC Q20, TA Instruments, New Castle, DE, USA) was used to evaluate the effect of the addition of Al on the onset melting and onset solidification temperature of the SAC105 solder. Each of the samples was weighed to approximately 10 mg and placed on an aluminum pan and covered with a lid. It was then heated from 25 °C to 300 °C and then cooled down to room temperature at a rate of 10 °C/min. For each composition, DSC test was repeated 3 times to ensure the reproducibility of the DSC results.

For reflow, commercial grade polycrystalline copper (Cu) sheets (30 mm × 30 mm × 0.3 mm) were used as substrates. Before soldering, Cu sheets were polished with 2000 Grit silica carbide paper, washed with detergent and soaked in 10 vol.% H_2_SO_4_ solution for 15–30 min to remove any oxide film present. These were then rinsed with distilled water followed by drying with acetone. Sparkle Flux WF-6317 (Senju Metal Industry, Tokyo, Japan) was then evenly placed on the Cu substrate. SAC-xAl thin disc (diameter = 6.5 mm, thickness = 1.24 mm) were placed in the middle of copper substrates which had been covered with Flux WF-6317. Reflow was carried out in an oven at 250 °C for 60 s. After the reflow process, the residual flux was cleaned and removed by rinsing the sample under running distilled water. After that, the reflowed samples were prepared by standard metallographic specimen preparation for microstructural investigation. The microstructure of the bulk solder and the morphology of the IMCs formed at the solder/substrate interface were investigated by FESEM and the composition of the IMCs was investigated by EDS. IMC thickness was calculated by dividing the IMC area by the length of the IMC using a built-in image analyzer software in an Olympus SZX10 (Olympus, Tokyo, Japan) stereoscope. For each experimental condition, thickness values were measured on 5 micrographs and the average IMC thickness is reported. For the top view observation of the interfacial IMCs, the solders were etched in a mixture of 93% CH_3_OH, 5% HNO_3_, and 2% HCl to remove the solder matrix and expose the interfacial intermetallic compound.

Vickers hardness measurement was performed to investigate the relationship between microstructure and microhardness. The Vickers hardness values were obtained as [36]:

(2) HV= 2Psinφ/2d

where φ is the indenter apex angle, P is the applied load and d is the average length of diagonals. The applied load and loading period are 1 kgf and 5 s, respectively. For each specimen, five points were tested and the mean values were obtained.

Nanoindentation testing was done using Hysitron Ubi-750 (Hysitron, Minneapolis, MI, USA). Two modes of indentation were conducted on the samples: Quasi-static and Continuous Dynamic Measurement. Quasi-static indentation was conducted with a maximum load of 500 μN, holding time of 2 s, loading rate and unloading rate of 16.67 μN/s and continuous dynamic measurement test was conducted with a maximum of 1000 μN, holding time of 2 s and a loading rate and unloading rate of 16.67 μN/s. For each element, 5 points were tested and the mean values were obtained. The load–displacement data obtained were analyzed using the method proposed by Oliver and Pharr [37] to determine the hardness and elastic modulus as functions of the displacement of the indenter. For quasi static nanoindentation testing, hardness (H) and reduced modulus (E) can be obtained. The elastic modulus E of the material being indented is related to the reduced modulus using the following equation [38]:

(3) $$E=\;\frac{1-v^{2}}{\frac{1}{E_{r}}-\frac{1-v_{i}^{2}}{E_{i}}}$$

where v is the Poisson’s ratio of the indented material (usually assumed to be 0.3 if unknown), and v_i_ and E_i_ are the Poisson’s ratio and elastic modulus of the indenter tip material, respectively. The elastic modulus E_i_ and Poisson’s ratio v_i_ of the Berkovich indenter used in this study are 1141 GPa and 0.07 respectively. For continuous dynamic measurement, results for the hardness and complex modulus as a function of indentation depth can be obtained.

Complex modulus, which is also known as dynamic modulus, is a ratio of stress strain under vibratory conditions. It could be defined by the equation below [39]:

(4) $$E^{*}=E^{\prime }+\text{i}E^{\prime \prime }$$

where E* is the reduced complex modulus, E′ is the reduced storage modulus (or elastic modulus), E″ is the loss modulus and i is the imaginary unit.

## 5. Conclusions

The following conclusions can be drawn from this study:

- Minor addition of Al has reduced the undercooling of SAC105 solder significantly.
- With addition of 0.1–0.5 wt.% Al to SAC105, Cu_3_Al_2_ IMC was found.
- Cu_3_Al_2_ IMC segregated near the edge of solder upon reflow as Al was added up to 0.3 wt.%.
- Minor alloying Al has reduced the thickness of interfacial Cu_6_Sn_5_ IMC significantly but do not alter the morphology of Cu_6_Sn_5_ IMC.
- Minor alloying Al has reduced the thickness of interfacial Cu_6_Sn_5_ IMC but has no significant effect on the thickness of Cu_3_Sn during isothermal aging.
- It is suggested that the influence of Al exert its influence on the interfacial reaction by hindering the flow of reacting species at the interface during reflow.
- Cu_3_Al_2_ IMC has higher hardness and elastic modulus than other microstructure in SAC105 + Al microstructures.

## Figures and Tables

**Table 1:** Onset of melting, onset of solidification and undercooling of SAC105, SAC105 + 0.1Al, SAC105 + 0.3Al and SAC105 + 0.5Al.

Solder	Onset of Melting (°C)	Onset of Solidification (°C)	Undercooling (°C)
SAC105	216.84 ± 0.50	200.12 ± 0.64	~17
SAC105 + 0.1Al	216.43 ± 0.38	215.84 ± 1.39	~0.6
SAC105 + 0.3Al	221.14 ± 0.36	217.74 ± 0.83	~3.4
SAC105 + 0.5Al	221.65 ± 1.02	219.17 ± 0.21	~2.48

**Table 2:** Hardness and modulus of Pure Sn, Cu_6_Sn_5_, and Cu_3_Al_2_.

Element/Compound	SAC105		SAC + 0.1Al	
	E (GPa)	H (GPa)	E (GPa)	H (GPa)
Sn	49.43 ± 7.93	0.29 ± 0.07	50.43 ± 4.86	0.23 ± 0.04
Cu_6_Sn_5_	92.25 ± 16.24	6.20 ± 0.59	100.99 ± 10.09	6.26 ± 1.07
Cu_3_Al_2_	–	–	170.08 ± 12.92	10.52 ± 1.71
